# Prevalence of diarrhoea and associated risk factors among children under five years old in Pader District, northern Uganda

**DOI:** 10.1186/s12879-020-4770-0

**Published:** 2020-01-13

**Authors:** Stephen Omona, Geoffrey M. Malinga, Robert Opoke, Geoffrey Openy, Robert Opiro

**Affiliations:** 1 Department of Biology, Faculty of Science, Gulu University, Laroo Division, Gulu, Uganda; 2 Department of Environmental and Biological Sciences, Faculty of Science and Forestry, University of Eastern Finland, Joensuu, Finland; 3 Department of Biology, Faculty of Science, Muni University, Arua, Uganda; 4 Department of Biosystems Engineering, Faculty of Agriculture and Environment, Gulu University, Laroo Division, Gulu, Uganda

**Keywords:** Bivariate, Logistic regression, Multivariate, Risk factor, Pajule

## Abstract

**Background:**

Diarrhoea remains a major cause of morbidity and mortality in children under 5 years in sub-Saharan Africa. Of the three East African countries, Uganda has the worst mortality rate in children < 5 years, with 22% of these deaths attributed to diarrhoea. For proper planning and implementation of control, an understanding of the prevalence and determinants of the disease is crucial. This study assessed the prevalence of diarrhoea and related risk factors among children < 5 years in Pajule Subcounty, Pader District in northern Uganda.

**Methods:**

A cross-sectional survey was conducted in April 2018, covering 244 randomly selected households having children < 5 years old in the study area. A semi-structured questionnaire was used to interview the households about diarrhoeal history in their children in the last 2 weeks preceding the survey, and on the risk factors predisposing children to diarrhoeal infections. Bivariate and multivariate logistic regression analyses with a 95% confidence interval and *p* < 0.05 was used to identify the risk factors associated with childhood diarrhoeal disease.

**Results:**

We found a prevalence of diarrhoea of 29.1% [95% CI (23.7–35.0)] among children < 5 years in Pajule Subcounty during the 2 weeks preceding the survey. Use of unprotected water sources, age of child caretaker, child weaning time and family size had significant associations with diarrhoeal morbidity.

**Conclusion:**

The prevalence of childhood diarrhoea among children < 5 years of age in rural settings of Pajule Subcounty was higher than the Ugandan national average. Use of unprotected water sources, age of child caretaker, child weaning time and family size were identified as predictors of diarrhoeal occurrence. These findings underscore the need for improving access to clean water and providing community health education as the best methods for fighting childhood diarrhoea in the study area.

## Background

Diarrhoea, defined as having unusually loose or watery stool that occur more frequently than usual within 24 h [1], remain among the most common causes of mortality and morbidity in children, particularly in low and middle-income countries. Worldwide, diarrhoea accounts for an estimated 3.6% of the global burden of disease, as expressed in disability-adjusted life years [2], and it is the leading killer, accounting for approximately 8% of all deaths among children < 5 years despite the availability of simple effective treatments [3]. Although the global mortality from diarrhoea has been declining over the past 25 years, the disease is still a major cause of mortality in children < 5 years of age in developing countries, contributing up to 21% of deaths [4].

In Uganda, diarrhoea is among the top four causes of morbidity in infants and young children [5]. The Uganda Demographic and Health Survey of 2016 reported that the prevalence of diarrhoea among children < 5 years in Uganda was 20% [6]. In 2017, diarrhoeal disease deaths reached 6.41% of total deaths, making the country to be ranked 27th worldwide [7]. Presently, diarrhoea still remains among the top ten causes of morbidity in the country, with rotavirus being responsible for about 40% of all diarrhoeal cases [8].

Pader District in northern Uganda was affected by the civil war between the Lord’s Resistance Army (LRA) and the Uganda People’s Defense Forces (UPDF) that plagued the region between the1980’s and 2008. This resulted in the creation of internally displaced persons’ camps (IDPs), disrupting social services delivery [9, 10]. Currently, the district lags behind the rest of the country in terms of the human development indices and is characterized by high levels of poverty [11]. Infant mortality rate [IMR] in the district is standing at a staggering 180+ per 1000 live births, with acute diarrhoea accounting for 8% of such deaths [12]. Sanitation remains a challenge with only 30% of the households having unimproved toilet facilities, and about 600,000 households do not have any toilet facility at all [13]. Despite these statistics, accurate information on prevalence and factors associated with diarrhoea in the district remain virtually unknown. The current study determined the prevalence of diarrhoea and risk factors among children < 5 years old with the view to provide information that could be useful in planning interventions to reduce the burden of the disease in the district.

## Methods

### Study area

**Fig. 1:**
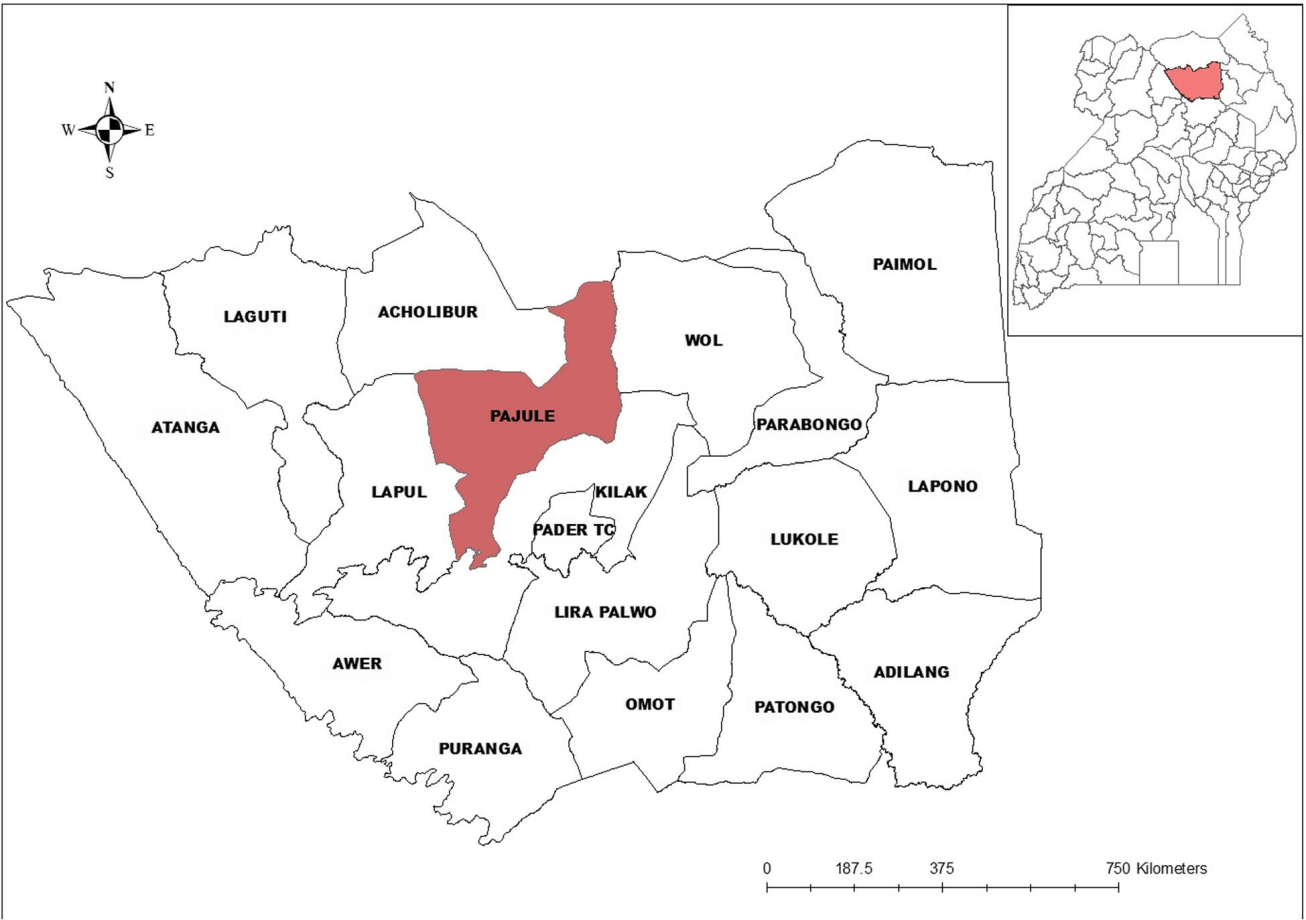
Location of the study area (Pajule Sub-county) in Pader District, northern Uganda. Map was created by the authors using ArcGIS version 10.3.1

The study was conducted in Pajule Subcounty (2^0^ 56′ 23″ N and 32^0^ 56′ 38″ E) located in Aruu North constituency, Pader District in northern Uganda [Fig. 1]. Pajule Subcounty consists of six parishes and has a population of 22,713, with 4050 of these being children below 5 years [11]. Like in other parts of northern Uganda, poverty level is higher than the national average, due to a combination of factors like the prolonged civil war that affected the entire northern region, cattle rustling by the Karimojong, and marginalisation that dates back to the colonial era [9]. The majority of the households derive their livelihood from subsistence farming; only 27% depend on earned income [13]. Water coverage has reduced from 57% when the population was in camps to only 38% as the communities returned to their homes [14].

### Study design and data collection

A cross-sectional survey was conducted in April 2018 in four randomly selected parishes out of the six parishes in Pajule Subcounty. According to the 2014 National Housing and Population Census, Pajule Subcounty has approximately 4000 households, and so a sample size of 351 households was estimated using the Krejcie and Morgan table [15]. However, due to logistical constraints, non-response or unavailability of targeted respondents at the time of survey, we only sampled a total of 244 households. The number of households in each parish was determined using probability proportional to size, and from each parish, at least two villages were randomly selected using a random number. The list of households in each village was obtained from the respective Local Council chairpersons. Only individuals from households where the mother or caregiver was present and had a child < 5 years old were interviewed. In cases, where there were more than one child < 5 years in the same household, index child was selected by lottery method. Trained research assistants administered semi-structured questionnaires based on the World Health Organization (WHO) guidelines [16]. The dependent or outcome variable was the presence of diarrhoea among children aged < 5 years within the 14 days before the survey. This was evaluated by asking the mother or caregiver if the child involved in the study had suffered from diarrhoea within 14 days before the study. Independent variables included socio-demographic, socio-economic, environmental and behavioural factors. Socio-demographic and economic characteristics included age of the child, number of children under the age of five in the household, family size, age of the child’s caregiver, sex of the child, the income status of the family, and the mother’s or caregiver’s education level. Environmental factors included type of water source, availability of animals in the homestead, presence of animals’ houses, child’s stool disposal practice, availability of latrines, ownership of latrine, hand washing practices, availability of kitchen, household’s environmental cleanliness, and presence of utensils’ drying racks. The behavioral characteristics included source of drinking water, boiling of water before consumption, frequency of warming cold food, weaning age, and age of food supplementation.

### Data analyses

Descriptive analyses using frequency and percentages were used to summarize the independent and dependent variables. To obtain the associations between diarrhoea among children and risk factors, we used multivariable logistic regression. The adjusted odd ratios [AORs] of having diarrhoea with 95% confidence interval [CIs] and *P* value < 0.05 were used to describe associations. First, we conducted univariate analyses to determine the associations between diarrhoea and other associated factors using chi-square and binary logistic regression. Eight variables with *p*-values less than 0.05 in bivariate analyses were included in the final multivariable logistic regression. All analyses were done using IBM SPSS for windows version 25.

## Results

### General characteristics of study households

Of the 244 households surveyed, 11.1% of the respondents were mothers or caretakers with no formal education while the majority (68%) had primary level education (Table 1), and 20.9% had secondary education. In terms of latrine coverage, 79.9% of the households had latrines in their homesteads while 68.9% reported sharing of latrines with other nearby households. For those who had latrines, only 14.3% had hand-washing facilities erected near the latrines. The practice of disposal of children’s stool was fairly well addressed with 80.7% of the respondents properly disposing of children’s stool as opposed to 19.3% who disposed of tools unsafely. According to the World Health Organization [17], a child’s stool is considered to be disposed of safely when he/she uses either the toilet or latrine and puts or buries the faeces in the toilet/latrine. Furthermore, majority (83.2%) of the households gave food supplements to children when aged > 6 months, and 49.2% weaned their children at the age of > 1 year. Majority (45.5%) of the respondents had a family size less than five individuals and only 63.5% of mothers/caretakers completed their immunization schedules as required (Table 1).

**Table 1:** General characteristics of the households/respondents surveyed. Significant *p* values in bold

Variable	n	Percentage	95% CI		χ^2^	p
Sex of the child			Lower	Upper		
Male	127	52.0	48.5	58.3	1.302	0.254
Female	117	48.0	41.7	54.2		
Age of child (months)						
0–12	69	28.3	22.9	34.2	4.216	0.378
13–24	98	40.2	34.2	46.4		
25–36	44	18.0	13.6	23.2		
37–48	16	6.6	4.0	10.2		
49–59	17	7.0	4.3	10.7		
Number of under five children						
Up to one	115	47.1	40.9	53.4	0.997	0.318
Two or more	129	52.9	46.6	59.1		
Caretaker						
Mother	216	88.5	84.1	92.1	0.902	0.342
Others	28	11.5	7.9	15.9		
Age of mother/child caretaker						
6 to 15	6	2.5	1.0	5.0	6.729	**0.035**
16 to 30	159	65.2	59.0	70.9		
≥ 31	79	32.4	26.7	38.4		
Education level of the mother or child caretaker						
No formal education	27	11.1	7.6	15.5	0.304	0.859
Primary	166	68.0	62.0	73.6		
Secondary and above	51	20.9	16.2	26.3		
Number of household member/family size						
Less than 5	111	45.5	39.3	51.8	10.763	**0.005**
6 to 9	94	38.5	32.6	44.7		
10 to 15	39	16.0	11.8	21.0		
Income status of the family						
Poor	225	92.2	88.3	95.1	0.559	0.439
Rich	19	7.8	4.9	11.7		
Source of drinking water						
Borehole	20	8.2	5.2	12.1	4.724	0.094
Piped water	56	23.0	18.0	28.5		
Wells	168	68.9	62.8	74.4		
Nature of water source						
Protected	147	60.2	65.4	76.7	23.339	**< 0.001**
Unprotected	97	39.8	23.3	34.6		
Houses shared with domestic animals						
No	26	10.7	7.2	15.0	4.349	**0.037**
Yes	218	89.3	85.0	92.8		
Disposal of the youngest child’s stool						
Proper way	197	80.7	75.4	85.3	1.411	0.235
Improper way	47	19.3	14.7	24.6		
Latrine availability						
Yes	195	79.9	74.6	84.6	0.930	0.335
No	49	20.1	15.4	25.4		
Ownership of latrine						
Shared	76	31.1	25.6	84.6	0.771	0.380
Private	168	68.9	62.8	74.4		
Environmental cleanliness						
Clean/safe	174	71.3	65.4	76.7	0.672	0.412
Unclean/unhygienic	70	28.7	23.3	34.6		
Handwashing facilities near the latrine						
Yes	35	14.3	10.4	19.2	1.640	0.200
No	209	85.7	80.8	89.6		
Availability of separate kitchen						
Yes	192	78.7	73.2	83.5	7.335	**0.007**
No	52	21.3	16.5	26.8		
Racks for drying utensils						
Yes	58	23.8	18.8	29.4	0.386	0.534
No	186	76.2	70.6	81.2		
Warming of cold foods						
Yes	149	61.1	54.8	67.0	17.221	**< 0.001**
No	95	38.9	33.0	45.2		
Boiling of drinking water						
Yes	14	5.7	3.3	9.2	3.470	0.062
No	230	94.3	90.8	96.7		
Age of child started supplementary food						
Less than 6 months	26	10.7	7.2	15.0	5.575	0.062
6–12 months	203	83.2	78.1	87.5		
> 12 months/not started	15	6.1	3.6	9.7		
Child weaning time						
On breastfeeding	109	47	38.5	50.9	7.420	**0.024**
Weaning < 1 year	15	6.1	3.6	9.7		
Weaning > 1 year	120	49.2	42.9	55.4		
Handwashing practices at critical times						
Yes	152	62.3	56.1	68.2	19.616	**< 0.001**
No	92	37.7	31.8	43.9		
Immunization status of child						
Yes	155	63.5	57.4	69.4	0.309	0.578
No	89	36.5	30.6	42.6		

### Prevalence of diarrhoea and associated risk factors among children

Overall, from a total of 224 households surveyed, 29.1% [95% CI (23.7–35.0)] reported episodes of diarrhoea in children < 5 years in the two-week period prior to data collection. The diarrhoeal prevalence in males, 52% [95% CI (48.5–58.3)] and females, 48.0% [95% CI (41.7–54.2)] did not differ significantly. Age group 13–24 months appeared most vulnerable, followed by 0–12 months, and the lowest prevalence was in category 37–48 months (Table 2). By age of caretaker, diarrhoea most commonly occurred among children whose mothers or caretakers were aged 16–30, 65.2% [95% CI (59.0–70.9)] and ≥ 31 years, 32.4% [95% CI (26.7–38.4)] than in those aged ≤15 years, 2.5% [95% CI (1.0–5.0)]. In the chi-square (Table 1) and univariable binary logistic regression analysis (Table 2), age of child caretaker, family size, nature of protection of drinking water source, availability of separate kitchen, child weaning time, warming of cold food, sharing of houses with domestic animals and mothers not washing hands at critical times had a *p*-value less than 0.05 and were further analyzed by multivariable logistic regression (Table 2). The multiple logistic regression revealed that the only factors significantly associated to diarrhoeal morbidity among children below 5 years in Pajule Subcounty are age of mother/child caretaker, family size, child weaning time and use of unprotected water sources such as wells (Table 2). Children whose mothers/caretaker were aged 16–30 years and ≥ 31 years had 14 times [AOR: 14.275, 95%CI (1.207–168.757)] and 12 times [AOR: 11.86, 95%CI (1.066–131.928)] higher odds of diarrhoea than those whose caretaker were aged less than 15 years. Children whose households had 10–15 children had seven times higher odds of diarrhoea than children whose households had one child [AOR: 7.185, 95%CI (1.353–38.147)]. The risk of developing diarrhoea in children whose households use protected water source had a 68% lower chance [AOR: 0.322, 95%CI (0.156–0.665)] compared to children in households who use unprotected water source (Table 2). Finally, children exclusively breastfed had 85% lower chance [AOR: 0.1542, 95%CI (0.034–0.595)] of diarrhoea than children who were weaned early (< 1 year).

**Table 2:** Multivariate logistic regression analyses of the risk factors associated with diarrhoea among children under five years in Pajule Subcounty, Pader District

Variables		Crude			Adjusted odds ratio, AOR		
	n	Odds ratio, OR	95% CI	*P* value	Adjusted odds ratio, AOR	95% CI	*P* value
Sex of the child							
Male	127	0.723	0.414–1.263	0.255			
Female	117	1					
Age of child (months)							
0–12	69	1					
13–24	98	0.804	0.412–1.568	0.522			
25–36	44	0.973	0.424–2.233	0.949			
37–48	16	2.857	0.594–13.736	0.190			
49–59	17	1.905	0.493–7.355	0.350			
Number of under five children							
Up to one	115	1					
Two or more	129	1.325	0.762–2.306	0.319			
Caretaker							
Mother	216	1					
Others	28	1.578	0.611–4.075	0.346			
Age of mother/child caretaker (years)							
6 to 15	6	1			1		
16 to 30	159	4.360	0.773–24.595	0.095	**14.275**	**1.207–168.757**	**0.035**
≥ 31	79	7.294	1.230–43.261	0.029	**11.860**	**1.066–131.928**	**0.044**
Education level of the mother or child caretaker							
No formal education	27	0.757	0.276–2.076	0.588			
Primary	166	0.930	0.462–1.874	0.840			
Secondary and above	51	1					
Number of household members/family size							
Less than 5	111	1			1		
6 to 9	94	1.214	0.676–2.181	0.516	0.934	0.431–2.024	0.863
10 to 15	39	6.500	1.880–3.895	0.003	**7.185**	**1.353–38.147**	**0.021**
Income status of the family							
Poor	225	1.467	0.553–3.895	0.441			
Rich	19	1					
Source of drinking water							
Borehole	20	1					
Piped water	56	0.200	0.042–0.952	0.043			NS
Wells	168	0.270	0.060–1.207	0.087			
Nature of water source							
Protected	147	1			1		
Unprotected	97	0.248	0.139–0.444	< 0.001	**0.322**	**0.156–0.665**	**0.002**
Houses shared with domestic animals							
No	26	1					
Yes	218	0.288	0.084–0.991	0.048			NS
Disposal of the youngest child’s stool							
Proper way	197	1					
Improper way	47	0.666	0.340–1.305	0.237			
Latrine availability							
Yes	195	1					
No	49	0.721	0.370–1.404	0.336			
Ownership of latrine							
Shared	76	0.769	0.428–1.383	0.380			
Privately	168	1					
Environmental cleanliness							
Clean/safe	174	1					
Unclean/unhygienic	70	0.778	0.427–1.418	0.413			
Handwashing facilities near the latrine							
Yes	35	1					
No	209	0.566	0.235–1.364	0.205			
Availability of separate kitchen							
Yes	192	1					
No	52	0.420	0.222–0.795	0.008			NS
Racks for drying utensils							
Yes	58						
No	186	0.810	0.416–1.576	0.535			
Warming of cold foods							
Yes	149	0.305	0.172–0.541	< 0.001			NS
No	95						
Boiling of drinking water							
Yes	14	1					
No	230	0.176	0.023–1.370	0.097			
Age of child started supplementary food							
Less than 6 months	26	1					
6–12 months	203	2.195	0.958–5.033	0.063			
> 12 months	15	5.571	1.042–29.780	0.045			
Child weaning time							
On breastfeeding	109	1			1		
Weaning < 1 year	15	0.242	0.079–0.738	0.879	**0.142**	**0.034–0.595**	**0.008**
Weaning > 1 year	120	0.956	0.533–1.714	0.015	1.140	0.549–2.366	0.726
Mothers’ handwashing practices at critical times							
Yes	152	1					
No	92	0.281	0.158–0.499	< 0.001			NS
Immunization status of child							
Yes	155	1					
No	89	0.579	0.660–2.105	0.579			

*NS* not significant

*OR* odd ratio

*AOR* adjusted odd ratio

*CI* confidence interval

Significant results are bold

## Discussion

We assessed the prevalence and risk factors of diarrhoea among children under 5 years old in Pajule Subcounty in Pader District, northern Uganda. Overall, the mothers or caretakers reported a prevalence of diarrhoea of 29.1%, which is lower than the 40.8% reported in neighboring Agago District [18], and elsewhere in Uganda; 41.3% in Adjumani Refugee Camp in West Nile [19] and 40.3% in Sembabule District [20]. However, the reported prevalence is higher than the 20% reported for the same age group in the Uganda Demographic and Health Survey of 2016 [6]. The high prevalence of diarrhoea in Pajule Subcounty than the national average could be due to the fact that the area suffered a prolonged conflict which disrupted social services like education and health, infrastructural development, and the overall economic fabric of the society [9]. For example, 11.1% of the mothers or caretakers had no formal education, and the majority (68.0%) had stopped in the lower primary (Primary one to four). Although this was not significant in our analyses, the role of formal education cannot be under-rated, as less educated people are less likely to take their hygiene and sanitation seriously, as well as those of their children.

Our results showed that family size, the age of child caretaker, child-weaning time, nature of protection of water source had significant associations with diarrhoeal morbidity. Children whose households had 10–15 children had seven times higher odds of diarrhoea than children whose households had one child. High number of individuals in a household potentially compromises hygiene and sanitation, making children more prone to contact with diarrhoeal pathogens. In Pajule, house sizes are mostly small temporary huts where humans, and sometimes pets, occupy the limited space, further reducing cleanliness in the household.

The present study also showed that exclusively breastfed children had 85% lower chance of diarrhoea than children who were weaned early (at less than 1 year). Given the poor hand washing practices and general unhygienic conditions observed in this study, preparations of weaning foods have the potential of spreading diarrhoeal causing germs to the infants. Weaning foods prepared under unhygienic conditions are frequently heavily contaminated with pathogens and are thus a major factor in the cause of diarrhoeal diseases and associated malnutrition [21]. Our results are consistent with many previous studies that have indicated that the addition of early food supplements to infants fed under prevailing environmental conditions in developing countries lead to their increased diarrhoeal attacks and associated reduced food intake [22].

Children whose caretakers were older had 12–14 times higher odds of diarrhoea than caretakers aged less than 15 years. This finding is surprising because previous studies have shown that young mothers are associated with a higher odds of diarrhoea than older mothers [23]. This is because, older caretakers tend to have experience in taking care of children compared to their younger counterparts and hence reducing childhood diarrhoeal incidences. Our results could however be explained by the fact that the majority of mothers were in teenage age (age 16–30) (Table 1).

Our study also showed that the risk of developing diarrhoea in children whose households use protected water sources was 68% lower compared to their counterparts who use unprotected water sources. This finding is similar with a study by [24] in Kenya who found that sources of drinking water was one of the household characteristics that had significant influence on childhood diarrhoea. However, a study conducted in southwest Ethiopia by [25] did not find any significant association of diarrhoeal occurrence and drinking water sources. Nevertheless, unprotected water sources have higher chances of fetching germs from the intruding animals or from running water carrying waste matters. In Pajule, like the rest of northern Uganda, access to safe water is a major challenge due to inadequate funding for construction of clean water sources and/or inadequate training of users in water source maintenance [26]. Due to lack of access to safe water, communities are forced to utilize unsafe sources such as streams, which requires boiling to make it safe.

## Conclusions

In the current study, prevalence of diarrhoea among under-five children in the rural setting of Pajule Subcounty in Pader was found to be high (29.1%). The use of unprotected water sources, age of child caretaker, child weaning time and family size had significant associations with diarrhoeal occurrence. These are mainly household level factors that can be mitigated by provision of access to clean water and community health education to fight childhood diarrhoea in the study area.

### Study limitations

Our study is prone to recall bias since it was based on respondents’ recalling of diarrhoeal history in their children within the last 2 weeks preceding the survey. However, we asked the mothers to report on the diarrhoea episode within 2 weeks from the time of the interview to reduce on recall bias. Additionally, diarrhoea prevalence was based on self-reported screening and was not further confirmed. Also, being cross-sectional in design did not take into account seasonal variation in the prevalence; data was collected in April 2018, which is the beginning of the wet season in northern Uganda. Follow up studies should cater for seasonal variation as well as stool and water analysis for the diarrhoeal causal agents and contaminations.

## Data Availability

The datasets used and/or analysed during the current study are available from the corresponding author on reasonable request.

## Abbreviations

AOR: Adjusted Odds Ratio
CI: Confidence Interval
IDPs: Internally Displaced Persons
IMR: Infant Mortality Rate
LRA: Lord’s Resistance Army
SPSS: Statistical Package for the Social Sciences
UPDF: Uganda People’s Defense Forces
WHO: World Health Organization
